# The hydrodynamic torque dipole from rotary bacterial flagella powers symmetric discs

**DOI:** 10.1038/s41567-026-03189-4

**Published:** 2026-03-27

**Authors:** Daniel Grober, Tanumoy Dhar, David Saintillan, Jérémie Palacci

**Affiliations:** 1 Institute of Science and Technology Austria, Klosterneuburg, Austria; 2 Department of Mechanical and Aerospace Engineering, University of California, San Diego, San Diego, CA USA

**Keywords:** Fluid dynamics, Physics, Biophysics

## Abstract

Swimming bacteria move through a fluid by actuating their moving body parts. They are force-free and can be described as hydrodynamic force dipoles: pushers or pullers. This modelling description is broadly used in biological physics and active matter research, and it has successfully predicted, for example, the superfluid behaviour of suspensions of pushers or the bend instability and emergence of turbulent flows in active nematics. However, this description accounts only for the translational motion of the swimming body and neglects the effects of hydrodynamic torque dipoles, which are relevant to bacteria with rotary motor-driven flagella, such as swimming *Escherichia* *coli*. Here we show that the torque dipole of confined swimming *E. coli* can power the persistent rotation of symmetric discs. The torque dipole leads to a traction force on the discs, an additive mechanism that is both contactless and independent of the orientation of the bacteria. Our results indicate that the torque dipole of swimming *E. coli* is notable in confined geometries, which is relevant to bacterial transport through porous materials, biofilms and the development of chiral fluids.

## Abstract (web summary)

It is known that placing asymmetric objects in a bacterial bath results in the net rotation of those objects. Now it is shown that the torque dipole of confined *E.coli* can rotate symmetric objects hydrodynamically.

## Main

Motile bacteria are micrometre-sized, self-propelling machines that convert biochemical energy from carbon and oxygen sources into mechanical work and motion. Owing to the kinematic reversibility of motion at low Reynolds numbers, the propulsion mechanism must break time-reversal symmetry to achieve a net displacement^1^. For *Escherichia* *coli* bacteria, this is achieved with the actuation of flagella by the proton-driven bacterial flagellar motor (BFM)^2^, whose persistent rotation propels the bacteria forward^3,4^. This results in the transfer of mechanical work from the bacteria to the fluid, which takes the surrounding medium out of equilibrium and is effectively an active bath. This leads, for example, to an increase in the diffusivity of colloidal spheres controlled by the activity of the bacteria^5–8^. Notably, in an apparent departure from equilibrium physics, bacterial baths have been used to power microgears, provided they have asymmetric shapes^9–11^. In these experiments^9,10^, symmetric gears simply fluctuate, whereas asymmetric gears display persistent rotation, whose direction is controlled by the geometry^12^. This strategy has been replicated with active colloids nested in asymmetric gears^13^ and used to control the propulsion of objects in bacterial baths^14–17^. The rotation in these examples results from the rectification of the forces exerted by the bacteria as they push against the walls, following pioneering work on bacteria tethered to a microrotor^18^.

Moreover, we recently demonstrated that colloidal aggregates exhibit persistent rotation when immersed in a suspension of swimming *E. coli*. We reported that the direction of rotation was controlled by the slip conditions of the interface where the aggregates sit. Colloidal aggregates rotate clockwise on the no-slip surface of a glass capillary and anticlockwise on an air–water interface, reminiscent of the change of direction of rotation of swimming *E. coli* on interfaces^19^. In effect, suspensions of swimming *E. coli* constitute chiral active baths that transmit torque to colloidal aggregates and, ultimately, control the formation of unconventional gels^20^.

**Fig. 1:**
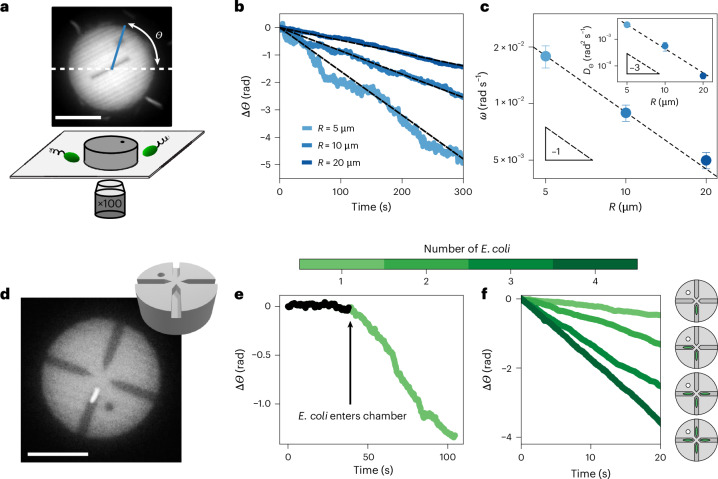
Dynamics of colloidal pucks in a bath of swimming *E. coli*. **a**–**c**, Collisions from *E. coli* swimming clockwise rotate symmetric discs (*ρ*_B_ = 6 × 10^8^ cells per millilitre). **a**, Bottom: schematic of the experiment. Nanoprinted discs (dubbed ‘pucks’) are immersed in a bath of swimming *E. coli* bacteria. Top: fluorescence microscopy image of a puck and the definition of its orientation *Θ*. **b**, Dynamics of pucks, *Θ*(*t*), for radii *R* = 5 μm (light blue), 10 μm (blue) and 20 μm (dark blue). All pucks exhibit a slow clockwise rotation. A linear fit gives the average rotation rate *ω* (dashed black lines). **c**, Puck rotation rate *ω*. Insets: rotational diffusivity *D*_Θ_ for pucks of difference sizes, measured from the mean squared angular displacement (Supplementary Information). The triangles indicate a slope of −1 (main panel) and −3 (inset). Data symbols with error bars represent the average and standard deviation for pucks with the same radius *R* = 5 μm (*N* = 6), 10 μm (*N* = 7) and 20 μm (*N* = 4). **d**–**f**, *E. coli* in confinement power symmetric discs (*ρ*_B_ = 3 × 10^7^ cells per millilitre). **d**, Fluorescence microscopy image of a puck with four chambers, each ending near the centre of the disc, as visible from the 3D design (inset). The chambers are 2 μm in height and 2 μm in width. A GFP-labelled *E. coli* is visible in the lower chamber. **e**, At the present concentration, the puck barely rotates until an *E. coli* enters the chamber, leading to a drastic increase in the clockwise rotation, which persists as long as the bacterium occupies the chamber. **f**, Dynamics of rotation Δ*Θ*(*t*) for pucks containing different numbers of trapped *E. coli* (see schematics on the right). The pucks all rotate clockwise, with rates increasing with the number of bacteria. Scale bars, 10 μm.

Colloidal aggregates, however, form from random collisions and, thus, lack the shape asymmetry required in refs. ^9,10,12^. This raises the question of whether bacterial baths can power the persistent rotation of objects in the absence of shape asymmetry. To investigate this, we leverage state-of-the-art three-dimensional (3D) nanoprinting and study the dynamics of symmetric microdiscs, dubbed ‘pucks’, immersed in suspensions of motile *E. coli* (Fig. 1a). We, thus, identify a new mechanism for the transduction of torque from the rotary motor-driven flagella towards objects in their surroundings. Here we show that the BFMs of swimming *E. coli* exert a torque dipole and a traction force that leads to the rotation of even symmetric objects. This hydrodynamic mechanism is contactless and relies on confinement rather than the shape asymmetry of the gears, thus contrasting with previous work on micromachines powered by bacteria or active colloids^9,10,12^. Our findings highlight the importance of the bacterial hydrodynamic torque dipole in confinement. The implications for dense and confined suspensions need to be investigated.

## Results

### Bacteria are torque dipoles and swim in circles

As our observations were performed near the bottom of a glass capillary, where objects are confined by gravity, we first recapitulate the swimming behaviour of swimming *E. coli* near a no-slip wall. Swimming *E. coli* are force- and torque-free. They are accurately represented hydrodynamically by a force dipole^21^ and a torque dipole: the flagella spinning one way and the body spinning the other way to balance the torque. In effect, swimming *E. coli* are hydrodynamically attracted to solid walls by the image charge of the force dipole^22^ and swim in (clockwise) circles as a result of the opposing shear forces induced by the torque dipole^23^ (Supplementary Fig. 2a).

### Experimental procedure

We now study the dynamics of 3D-printed discs, dubbed ‘pucks’, in the presence of swimming bacteria. The pucks were printed with radius *R* = 5 μm, 10 μm or 20 μm and constant height ~6 μm using a two-photon-polymerization printer (NanoOne, Upnano) (Fig. 1a and Supplementary Information). After printing and development, they were dispersed in a solution of 5% F 108 surfactant to prevent aggregation and were subsequently concentrated (Supplementary Fig. 1). The pucks were added to a suspension of swimming *E. coli* in a motility medium and sealed in a glass capillary (Methods). The concentration of swimming *E. coli* (*ρ*_B_) was adjusted before the experiment and is described in each section. The pucks sedimented. They sat at the bottom of the capillary and interacted with swimming *E. coli* (Fig. 1a). We carried out our observations by fluorescence microscopy using the autofluorescence of the nanoprinting resin and the green fluorescent protein (GFP) tag of the bacteria (Supplementary Information). A dot and a line were added to the design so that we could track the orientation *Θ*(*t*) of the pucks (Fig. 1a).

### Collisions with *E. coli* swimming clockwise rotate symmetric discs

We first observed the dynamics of simple pucks—thick discs—in a bacterial bath of concentration *ρ*_B_ = 6 × 10^8^ cells per millilitre. The clockwise rotation of aggregates was observed in ref. ^20^. Bacteria did not cross underneath the discs, as visible from fluorescence microscopy. They collided with the perimeter of a puck and deflected (Supplementary Video 1 and Supplementary Fig. 2b). The discs exhibited noisy dynamics at short times, reminiscent of the high effective temperature of the bacterial bath^5–7,20^ (Fig. 1c inset and Supplementary Fig. 3). Over the course of minutes, the pucks displayed a slow but perceptible clockwise rotation for all tested radii *R* = 5 μm, 10 μm and 20 μm (Fig. 1b). We quantified these observations by tracking the angle *Θ*(*t*) (Fig. 1a) and computing the rotation rate *ω*_R_ of the pucks from a linear fit of *Θ*(*t*). We found that *ω*_R_ ∝ 1/*R* (Fig. 1c), as previously found for colloidal aggregates in bacterial baths^20^. In brief, the curved trajectories of the swimming bacteria, as exemplified in Supplementary Fig. 2, lead to asymmetric collisions with the puck. These collisions produce a net torque and persistent rotation, in the absence of shape asymmetry (see ref. ^20^ for details of this toy model and Supplementary Section 2). The rotation is driven by forces on the perimeter of the disc, a mechanism akin to conventional bacterial machines (for example, see refs. ^9,10,12,16,17^), whereby a shape asymmetry was used to rectify the motion of *E. coli* and power rotation. In the present experiment, the asymmetry arises from the chirality of the clockwise trajectories of the *E. coli* swimming above the solid interface of the glass capillary.

We estimate the effective tangential force F^*^ resulting from collisions of the puck with swimming bacteria as ${F}^{* }=\frac{\omega }{{M}_{\Theta }R}\approx$ 0.006–0.06 pN, based on the reported rotation rate *ω* ≈ 10^−3^–10^−2^ rad s^−1^ (Fig. 1c) and independent measurements of the rotational mobility *M*_*Θ*_ of the puck (Supplementary Fig. 4). This value of F^*^ is markedly smaller than the effective pushing force per cell measured for micromotors powered by swimming *E. coli*, *F* ≈ 0.2 pN (ref. ^12^), as well as the typical flagellar thrust of *E. coli* cells^21,24^. This reflects the minimal rectification proportional to *ℓ*_B_/*R*_c_ arising from the collisions of the curved trajectories with the puck perimeter, where *R*_c_ ≈ 50 μm is the radius of curvature of the trajectories and *ℓ*_B_ the bacteria length, as discussed in Supplementary Section 2. The effect is, however, sufficient to drive persistent rotation over long timescales and control unconventional aggregation^20^. Notably, our simple model satisfactorily predicts the observed rotation rate when accounting for the collision rate observed in the experiment (Supplementary Section 2).

### *E. coli* in confinement power symmetric discs

**Fig. 2:**
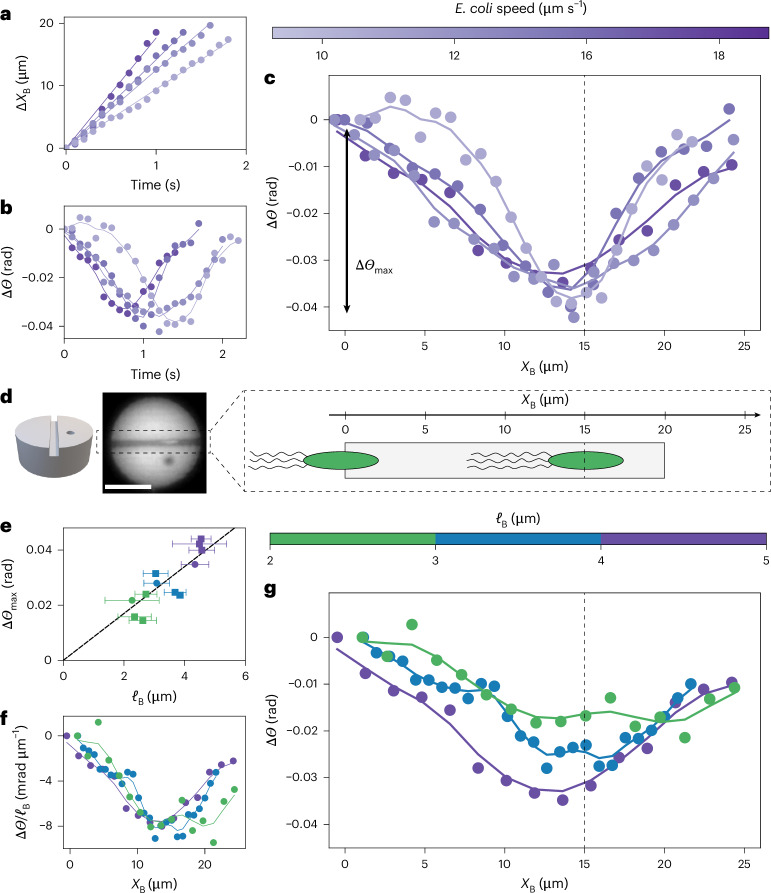
Dynamics of pucks with a single swimming *E. coli* crossing the channel. **a**, Position of swimming *E. coli* (*X*_B_(*t*)) inside the channel. Different colours indicate different bacteria. The velocity is obtained by a linear fit (solid lines). **b**, Rotation of the puck, Δ*Θ*(*t*) = *Θ*(*t*) − *Θ*(0), as a single swimming *E. coli* passes through the channel. Different colours indicate different bacteria with different velocities (colour bar). **c**, The curves Δ*Θ*(*t*) from **b** collapse when represented as Δ*Θ*(*X*_B_), as prescribed by low-Reynolds-number dynamics. Δ*Θ*(*X*_B_) decreases before reversing direction, presenting a characteristic ‘down–up’ shape. The dashed line highlights the location of the minimum: *X*_B_ = 2*R* − *ℓ*_B_ ≈ 15 μm. The depth of the down–up shape is denoted Δ*Θ*_max_. **d**, Left: schematic of a puck with a single channel. Middle: image of a puck. Right: schematic representation of *E. coli* swimming through the channel. The body and flagella are for a puck of radius 10 μm. The dashed line is the dashed line in **c**. **e**, Depth of the dip, Δ*Θ*_max_, as a function of the body length of *E. coli* (*ℓ*_B_). Each data symbol represents a crossing event by a bacterium (*N* = 12 events). Error bars represent the standard deviation for the bacteria length (*ℓ*_*B*_) determined by fluorescence microscopy. **f**, Plot of Δ*Θ*(*X*_B_)/*ℓ*_B_ showing the collapse of the first part of the down–up shape, as predicted by the model. **g**, Rotation of the puck, Δ*Θ*(*X*_B_), for *E. coli* of different sizes (*ℓ*_B_). The data do not collapse, in contrast to bacteria moving at different speeds (**c**). For larger *E. coli*, the minimum is deeper and occurs earlier. In all panels, solid lines are a Gaussian extrapolation of the experimental data (solid dots). Scale bar, 10 μm.

In this section, we present a new type of bacterial machine, one that is powered by the torque dipole of individual *E. coli* confined beneath symmetric discs. The effect is contactless, as it does not have the aforementioned collisions that power conventional bacterial ratchets. Here we present the experimental evidence that led us to unveil this new physical mechanism. We introduce two variants of the circular pucks, each with fixed radius *R* = 10 μm. Our aim was to confine individual *E. coli* underneath them. The first kind is a disc with four narrow chambers, placed radially, each terminating near the centre of the puck (Fig. 1d). The second kind has a single narrow channel, open on both ends, along the diameter of the puck (Fig. 2d). We present observations only of pucks where the chambers or channel lie on the bottom substrate, facing down. For the quantitative observations, we suspended the pucks in a dilute bacterial bath (*ρ*_B_ = 3 × 10^7^ cells per millilitre) and investigated their dynamics as they interacted with individual *E. coli*. Time-lapse data were acquired by spinning-disc confocal fluorescence microscopy (Nikon TI-2 Eclipse, 10 frames per second; Methods). These data were analysed to record simultaneously the position of the centre of mass of the puck, its orientation *Θ* and the position of an *E. coli* body confined beneath the puck. Note that only the body of each bacterium was fluorescently labelled and that the flagellum, a floppy tail of length ~6.5 μm (ref. ^25^), is not visible in the experiments.

We begin by describing the dynamics of the pucks with chambers following the entry of an *E. coli* into the chamber. The puck has four chambers, each with a square cross section (2 μm × 2 μm), placed radially and ending at a distance *d* ≈ 0.5 μm from the centre of the disc (Fig. 1d). The chambers are large enough for an *E. coli* to enter but too narrow for it to reverse direction; effectively, the *E. coli* becomes confined underneath the puck, while its body and flagella continue to spin. As soon as an *E. coli* positions itself in a chamber, the rotation rate of the puck increases drastically to *ω* ≈ 3 × 10^−2^ rad s^−1^, while always remaining clockwise (Fig. 1e). This was an unexpected finding as the chambers were designed to be radially aligned, thus suppressing any contribution to the torque from direct collisions with the walls. A simple estimate of the rotation rate of a puck arising from a single *E. coli* pushing against the wall of the chamber with force *F* gives *ω* = *M*_*Θ*_*Fd* (where the lever arm *d* ≈ 0.5 μm is the distance from the dead-end wall of the chamber to the centre of the puck). Thus, *ω* ≈ 2 × 10^−3^ rad s^−1^, an order of magnitude lower than observed in our experiments. We observed marked increases in the rotation rate each time another *E. coli* cell entered one of the other chambers (Fig. 1f). Notably, the fastest rotation rate was observed when there was a bacterium in each of the four chambers (Supplementary Video 2), a situation that should lead to stalling due to the bacteria pushing symmetrically. These results run contrary to previous reports of machines powered by bacteria or active colloids pushing on walls^9,10,14–17^ and require further research.

To elucidate the interplay between bacterial swimming and confinement, we investigated the model situation consisting of a single swimming *E. coli* crossing a puck through an open channel running along its diameter, in the absence of any collisions with the puck perimeter (Supplementary Video 3). In this design (Fig. 2d), there is no wall at the end of the chamber, eliminating the possibility of the bacterium pushing on the end wall. We focused on square channels with cross section 2 μm × 2 μm (Fig. 2), like the chambers used previously. Another geometry with a rectangular cross section is presented in Supplementary Fig. 5.

Swimming bacteria entered the channel and proceeded to exit the puck. The tight confinement prevented them from reversing course. Initially when a bacterium entered the channel, the puck rotated clockwise before eventually reversing direction, leading to a characteristic down–up shape in the dynamics of the puck orientation *Θ*. Notably, this down–up shape did not reverse when a bacterium entered from the other end of the channel (Supplementary Fig. 6). This result shows that the rotation of the puck—always clockwise when the bacterium entered the channel and anticlockwise as it exited—was not set by the direction of navigation of the bacterium.

We quantify our experimental observations by representing the change of angle Δ*Θ* of the puck after entry of the bacterium in the channel as a function of the position *X*_B_ of the centre of mass of the body of the bacterium in the channel. This representation allowed us to collapse data from bacteria with different swimming velocities (Fig. 2a–d), as expected from low-Reynolds-number dynamics. Indeed, the instantaneity of the Stokes equations^1^ dictates that the net motion of the puck is independent of the rate, that is the velocity, at which bacteria cross the channel. In effect, although bacteria with different swimming speeds (but the same body length, see below) (Fig. 2a) cross the channel in different times (Fig. 2b), the dynamics of the puck collapses when represented as a function of the position of the bacterium in the channel, Δ*Θ*(*X*_B_) (Fig. 2c), with a minimum at *X*_B_ ≈ 15 μm (black dashed line in Fig. 2c). Although the Δ*Θ*(*X*_B_) representation effectively collapses the dynamics of rotation of the pucks for similarly sized bacteria with different swimming velocities (Fig. 2a–c), there are noticeable differences in the depth (Δ*Θ*_max_) and position of the minimum for bacteria of different lengths (*ℓ*_B_) (Fig. 2g). Those differences do not correlate with the average angle of the cell body with respect to the channel (Supplementary Fig. 7). Instead, the depth of the dip (Δ*Θ*_max_) correlates with the size of the bacterium body (*ℓ*_B_) (Fig. 2e,f), as quantified by fluorescence imaging of the body. When bacteria with a longer body cross the channel, the dip is more pronounced (with larger Δ*Θ*_max_), and the reversal of direction occurs earlier (*X*_B_ is further from the exit).

### Hydrodynamic model

**Fig. 3:**
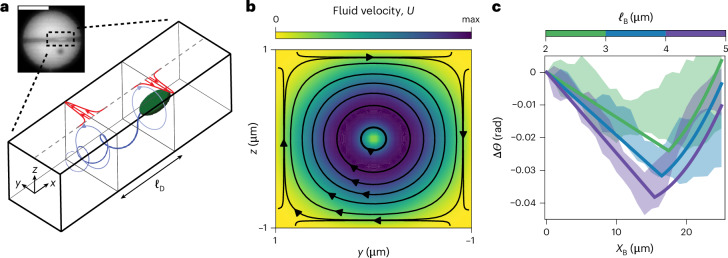
Hydrodynamic model of swimming *E. coli* passing through a channel. **a**, Sketch of *E. coli* swimming through a channel. Swimming *E. coli* exert a torque dipole on their surroundings, which stems from the counter-rotation of the cell body (clockwise when viewed from the rear) and flagella (anticlockwise). These two torques drive a hydrodynamic flow resulting in traction forces on the top wall of the channel (red arrows). The traction fields induced by each torque are offset by a distance *ℓ*_D_ and, thus, produce a net torque on the puck that drives the observed clockwise rotation. Inset: fluorescence microscopy image of an *E. coli* swimming through a channel. **b**, Hydrodynamic flow field from a single, clockwise-rotating rotlet near the front of the bacterium. **c**, Predictions of the model for bacteria of different lengths and, hence, dipole separation *ℓ*_D_ (solid lines) and comparison with the experimental measurements. The shaded zones represent the standard deviation of experimental measurements for four trajectories of each size of bacterium. The hydrodynamic model quantitatively captures the experimental observations. Scale bar, 10 μm. max, maximum.

The aforementioned observations rule out collisions of the bacteria with the inner channel walls—the driving force behind the rotation of asymmetric gears^9,10^—as a potential mechanism for the rotation of the pucks with a channel. Instead, we recall that swimming *E. coli* cells exert a torque dipole on their surroundings, which stems from the counter-rotation of the cell body (clockwise when viewed from the rear) and flagella (anticlockwise), and we intuit that these applied torques lead to the observed phenomenology. The rotation of the body entrains the fluid around it, resulting in a traction field (shear stress) on the walls of the channel. The counter-rotation of the flagella similarly generates an oppositely directed traction field. Because they oppose each other, the two traction fields do not result in a net force on the puck; however, as they are displaced along the channel axis by the effective length of the torque dipole *ℓ*_D_ (a distance of the order of the bacterial length), they can apply a net torque to the puck and drive its rotation (Fig. 3a).

To confirm this mechanism, we modelled the hydrodynamic interaction of a single *E. coli* cell swimming through a square microchannel of width 2*W*. The bacterium is assumed to be aligned with the axis of the channel, consistent with experimental observations. For analytical progress, we approximated the channel walls as infinite stationary boundaries. To leading approximation, the bacterium exerts both a force dipole and a torque dipole on the fluid around it. For a bacterium aligned with the channel axis (*x* direction), symmetry precludes the force dipole from driving any net torque, and we, therefore, omit it in our flow calculation. Instead, we idealize the bacterium as exerting two equal and opposite point torques (or rotlets) $\pm {\varGamma }_{{\rm{M}}}\,\hat{{\bf{x}}}$ at locations **r**_1,2_ offset by a fixed distance *ℓ*_D_ along the channel axis: ${{\bf{r}}}_{1}-{{\bf{r}}}_{2}={\ell }_{{\rm{D}}}\,\hat{{\bf{x}}}$. The microscopic torque magnitude *Γ*_M_ is given by the bacterial motor torque. The torque spacing, or dipole length *ℓ*_D_, is expected to scale with the size of the bacterium, a point we elaborate on below.

We first analyse the effect of a single rotlet $+{\varGamma }_{{\rm{M}}}\hat{{\bf{x}}}$ at location **r**_1_. At low Reynolds number, the fluid motion it induces inside the channel satisfies the Stokes equations,

1 $${\rm{\nabla }}\cdot {\bf{U}}=0,\,\,{\rm{\nabla }}\cdot \mathit{\varSigma} =-\frac{{\varGamma }_{{\rm{M}}}}{2}{\rm{\nabla }}\times [{\rm{\delta }}({\bf{r}}-{{\bf{r}}}_{1})\hat{{\bf{x}}}],$$

where **U** is the fluid velocity, *Σ* = −*PI* + 2*μE* is the Newtonian stress tensor expressed in terms of the pressure *P*, dynamic viscosity *μ* and rate-of-strain tensor $E=\frac{1}{2}({\rm{\nabla }}U+{\rm{\nabla }}{U}^{{\rm{T}}})$, and *δ*(**r**) is the Dirac delta function. The fluid velocity is subject to the no-slip condition at the channel walls: **U**(*x*, *y* = ±*W*, *z* = ±*W*) = **0**, where the *x* coordinate is aligned with the channel axis and the *z* direction is normal to the bottom substrate (Fig. 3a). By linearity of the Stokes equations, the fluid velocity depends linearly on the torque,

2 $${\bf{U}}({\bf{r}})=\frac{1}{8{\rm{\pi }}\mu }{{R}}({\bf{r}}-{{\bf{r}}}_{1})\cdot {\varGamma }_{{\rm{M}}}\hat{{\bf{x}}},$$

where *R*(**r**) is the Green’s function for this problem. As explained in Supplementary Information, the solution for **U**(**r**) can be obtained numerically by solving equation (1) using the boundary-element method^26^ (Fig. 3b). The velocity field in equation (2) exerts a traction on the channel walls. As the bottom wall is part of the fixed substrate, only viscous stresses on the top wall (*z* = +*W*) contribute to the vertical torque on the puck. There, the viscous traction is

3 $${\bf{t}}({\bf{r}})=-\hat{{\bf{z}}}\cdot 2\mu {{E}}({\bf{r}})=-\mu \left(\frac{{\rm{\partial }}{U}_{x}}{{\rm{\partial }}z}\hat{{\bf{x}}}+\frac{{\rm{\partial }}{U}_{y}}{{\rm{\partial }}z}\hat{{\bf{y}}}\right),\,\,\,z=+W.$$

This results in a net torque on the puck:

4 $${\varGamma }_{1}\hat{{\bf{z}}}={\int }_{\,z=+W}({\bf{r}}-{{\bf{r}}}_{{\rm{C}}})\times {\bf{t}}({\bf{r}}-{{\bf{r}}}_{1})\,{\rm{d}}S=-({x}_{1}-{x}_{C}){\int }_{\,z=+W}\mu \frac{{\rm{\partial }}{U}_{y}}{{\rm{\partial }}z}\,{\rm{d}}S\,\hat{{\bf{z}}}\,,$$

where **r**_C_ denotes the centre of the puck. Upon inserting equation (2), the torque magnitude reduces to

5 $${\varGamma }_{1}=-{{\varLambda}} \left(\frac{{x}_{1}-{x}_{C}}{W}\right){\varGamma}_{{\rm{M}}},\,\,\,\,{\rm{w}}{\rm{h}}{\rm{e}}{\rm{r}}{\rm{e}}\,\,\,\,{{\varLambda}} =\frac{W}{8\pi }{\int }_{\,z=+W}\frac{{{\partial }}{R}_{{yx}}}{{{\partial }}z}\,{\rm{d}}S.$$

This expression captures the transmission of the viscous torque from the point rotlet (*Γ*_M_) to the puck (*Γ*_1_). Note that *Λ* is a positive dimensionless constant independent of any parameters (including *W*); our boundary-element calculations in an infinite square channel provide a value of *Λ* ≈ 0.17.

As the bacterium swims through the channel, the torque dipole resulting from the counter-rotation of the cell body and flagella produces a net torque on the puck:

6 $$\varGamma \hat{{\bf{z}}}={\varGamma }_{1}\hat{{\bf{z}}}+{\varGamma }_{2}\hat{{\bf{z}}}=-\mathit{\varLambda} \frac{{\ell }_{{\rm{D}}}}{W}{\varGamma }_{{\rm{M}}}\hat{{\bf{z}}}\,,$$

where *ℓ*_D_ = *x*_1_ − *x*_2_ is the dipole length. Notably, the torque magnitude *Γ* is independent of the position of the bacterium under the puck, provided that both rotlets are inside the channel; it is also independent of the orientation of the bacterium ($+\hat{{\bf{x}}}$ or $-\hat{{\bf{x}}}$) along the channel axis, as observed in the experiment (Supplementary Fig. 6).

We can now describe the angular dynamics of the puck. We denote by *X*_B_(*t*) = *U*_s_*t* the instantaneous position of the bacterium inside the channel, measured from the channel entrance. Here, *U*_s_, the bacterial swim speed, is constant, as measured experimentally (Fig. 2a). If both rotlets are contained inside the channel, the torque on the puck is constant and given by equation (6), resulting in the angular velocity:

7 $$\frac{{\rm{d}}{\varTheta}}{{\rm{d}}t}={M}_{{\Theta }}\varGamma ,$$

where *M*_*Θ*_ is the rotational mobility of the puck for rotation around the *z* axis. The value of *M*_*Θ*_ is obtained from the Stokes–Einstein relation, *M*_*Θ*_ = *D*_*Θ*_/*k*_B_*T*, where *k*_B_ is the Boltzmann constant and *T* is temperature. The rotational diffusivity of the puck in a thermal bath was measured independently: ${D}_{\mathit{\varTheta} }=(6\pm 1)\times 1{0}^{-5}\,\,{\rm{r}}{\rm{a}}{{\rm{d}}}^{2}\,{{\rm{s}}}^{-1}$ (Supplementary Fig. 4). Integrating equation (7) and eliminating time using the swim speed provides the angular displacement as a function of the position *X*_B_ of the bacterium in the channel:

8 $$\Delta {\varTheta} ({X}_{{\rm{B}}})=-{\varLambda} \frac{{\ell }_{{\rm{D}}}}{W}\frac{{M}_{{\Theta} }}{{U}_{{\rm{s}}}}{\varGamma }_{{\rm{M}}}{X}_{{\rm{B}}}.$$

This relation predicts clockwise rotation of the puck and captures the linear decrease observed in the experimental data (Fig. 2c,g). All the prefactors in equation (8) can be estimated based on experiments (Supplementary Table 1), with the exception of the dipole length *ℓ*_D_. The collapse of the angular displacements upon scaling Δ*Θ* with cell body length in Fig. 2f points at a linear relation between *ℓ*_D_ and *ℓ*_B_, and therefore, we posit that *ℓ*_D_ = *αℓ*_B_. The dimensionless parameter *α* is the only fitting parameter in our model. By fitting equation (8) to the experimental data, we estimate *α* ≈ 1.5.

This simple hydrodynamic model allows us to explain the anticlockwise rotation of the puck, as observed in the second half of the down–up shape (Fig. 2c,g). As the cell body exits the channel, it ceases to exert a torque on the puck, which is now only subject to the torque *Γ*_2_ due to the rotating flagella, thus causing a change in the direction of rotation. We estimate the angular displacement beyond that point to be

9 $$\Delta \mathit{\varTheta} ({X}_{{\rm{B}}})= \frac{{\varLambda}}{W}\frac{{M}_{{\varTheta} }}{{U}_{{\rm{s}}}}{\varGamma }_{{\rm{M}}}\left[\frac{{X}_{{\rm{B}}}^{2}}{2}+{X}_{{\rm{B}}}({\ell }_{{\rm{B}}}-{\ell }_{{\rm{D}}}-R)+\frac{{\ell }_{{\rm{B}}}({\ell }_{{\rm{B}}}-2R)}{2}\right],$$

which predicts a reversal in the direction of rotation with a quadratic dependence on position. The hydrodynamic model provides a quantitative description of the rotation through equations (8) and (9). The rotation is controlled by either the two rotlets or a single one inside the channel. The transition is observed in the experiment as the position of reversal of the down–up shape (Fig. 2c).

## Discussion

We now inspect the physical implications of this hydrodynamic mechanism. First, the entrainment of the puck by a torque dipole explains the characteristic down–up shape observed in the experiment: when both rotlets are inside the channel, the puck rotates clockwise, and the direction of rotation reverses when a single rotlet remains in the channel. In effect, the model quantitatively reproduces our experimental findings (Fig. 3c) by capturing both the reversal of the direction of rotation and the depths Δ*Θ*_max_ observed in the experiment. In the experiment, the minimum, corresponding to the reversal of the direction of rotation of the puck, is observed near *X*_B_ ≈ 2*R* − *ℓ*_B_ (Fig. 2d). Naively, we expected the reversal of direction and position of the minimum to occur at *X*_B_ = 2*R* when the centre of the body of the *E. coli* exits the channel. We highlight two important simplifications in our far-field model that could explain this discrepancy and will require further work. First, the flagella form a deformable bundle of proteins whose complex dynamics are not fully captured by the point rotlet hydrodynamic description and may be affected by the geometric confinement. Second, we considered infinite channels in the model, whereas a near-field description might be required to fully describe the experimental results, especially at the point where the *E. coli* exits the channel. Nonetheless, the experimental observations confirm our intuition that the dipole length is linearly dependent on the size of the bacterium body *ℓ*_D_ = *αℓ*_B_, with *α* = *O*(1), and our experiments are well captured by the model with a single parameter *α* = 1.5.

**Fig. 4:**
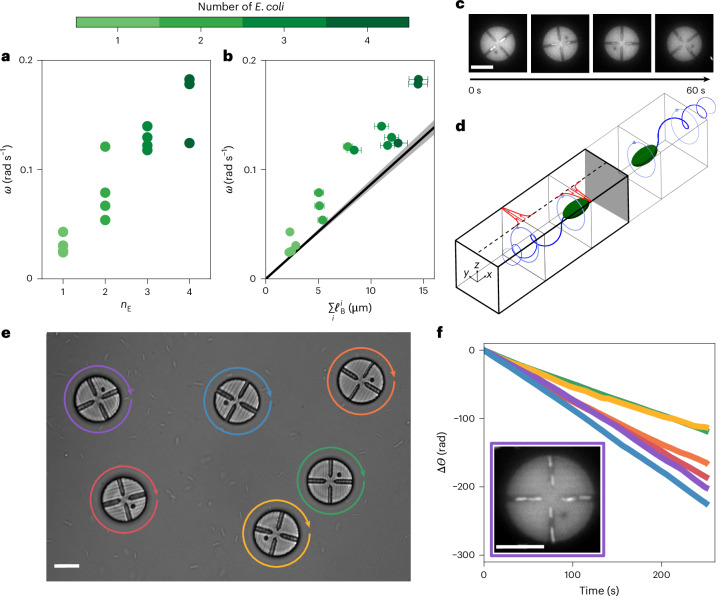
Dynamics of pucks with chambers. **a**, Rotation rates *ω* for pucks with chambers, as seen in Fig. 1d, for different occupancy levels *n*_E_ of the bacteria in the chambers. For all occupancy numbers (*n*_E_ > 0), the pucks rotate clockwise, with *ω* increasing with the number of bacteria in the chambers. This highlights the cumulative effect of the entrainment mechanism. **b**, The data from **a** (*ω*(*n*_E_)) collapse when *ω* is plotted as a function of the total body length inside the channels (${\sum }_{i}{\ell }_{{\rm{B}}}^{i}$), as predicted by the model. The black line indicates the prediction from the hydrodynamic model, which accounts for the hydrodynamics of a closed chamber rather than an infinite channel, as visible in **d**. Data symbols with error bars are centred on the value (*ω*) from **a** with a standard deviation from the determination of the bacterial length (*ℓ*_B_) using fluorescence microscopy. **c**, Time-lapse fluorescence microscopy images of a puck with one *E. coli* bacterium trapped inside each chamber. **d**, Schematic of the hydrodynamic model for the closed chamber, instead of the infinite channel in Fig. 3. The no-slip condition at the end of the chamber is implemented by placing an image system symmetrically on the other side of the wall. **e**, Experiment with six pucks with chambers suspended in a bath of motile *E. coli*. All pucks spin clockwise, marking a first step towards the development of chiral fluids of spinners. **f**, Dynamics Δ*Θ*(*t*) for each puck from **e**. The pucks rotated persistently for minutes at speeds up to 10 rpm. Inset: fluorescence microscopy image of a puck with four closed channels, each accommodating several *E. coli*, which rotated faster. Scale bars, 10 μm.

We now return to our experimental observations of pucks with chambers (Fig. 1d) in light of our mechanistic understanding. The hydrodynamic model presented in the section ‘Hydrodynamic model’ shows that when both the body and flagella of the *E. coli* are confined beneath the puck, they exert a torque on the puck, leading to persistent clockwise rotation. This torque always rotates the puck clockwise, regardless of the precise orientation of the cell inside the chamber; this explains the behaviour previously reported, whereby the puck rotates faster as other chambers are filled (Fig. 1f). Guided by our hydrodynamic model, we realized that the total length of trapped bacteria, rather than their number, should set the rotation rate, and we collapsed the observed rotation rates of the puck $\omega \propto {\Sigma }_{i}{\ell }_{{\rm{B}}}^{i}$, where ${\ell }_{{\rm{B}}}^{i}$ is the measured length of the body of bacterium *i* in the chamber (Fig. 4a,b). Furthermore, by simply modifying the hydrodynamics of an open channel to a closed one, our hydrodynamic model accurately predicts the observed rotation speeds (Fig. 4d). The clockwise rotation persists over the course of minutes at speeds comparable with asymmetric ratchets in bacterial baths^9,10^. This provides a way to form chiral fluids of spinners made of hybrid *E. coli*, micromachines that persistently rotate in the absence of geometric asymmetry. As a first step in this direction, we show a collection of discs powered by a bacterial bath (Fig. 4e and Supplementary Video 4). The rotation could be tuned by modifying the number of channels. The spinners can be fabricated with commercially available two-photon-polymerization nanoprinters. Notably, our experimental results highlight that capturing several bacteria inside narrow chambers (Fig. 4f inset) increases the rotation rate up to 10 rpm (Fig. 4f), which again demonstrates the salient role of confinement in enhancing the hydrodynamic effect.

## Conclusion

We unveiled a new mechanism for transducing the mechanical work of rotating flagellar nanomotors into the rotation of symmetric objects. The mechanism bridges orders of magnitude of scales, as confinement couples to the hydrodynamic torque dipole of swimming *E. coli* to power objects adjacent to the bacteria. Beyond the conceptual advance, our findings show powerful differences in comparison with the mechanism of rotation of asymmetric gears by rectification of flagellar thrust, as previously reported, for example, in refs. ^9,10,12^. Because the rotation emerges from confinement and the chirality of the BFM, it is agnostic to the shape of the object or the careful positioning of the bacteria underneath it. In practice, our results show that the torque dipole of organisms with rotary flagella should be important in a broad range of confinement situations, whether in natural, ecological or artificial settings. In light of the success of applying the force dipole to the description of biological systems and active matter^27–31^, the fluid mechanics implications of our findings remain to be explored. Our findings highlight the interplay between bacterial navigation and their environments, which has potential ecological implications, and they also reveal a new mechanism for assembling unconventional gels in bacterial baths^20^. Finally, our results provide a way to characterize aspects of individual bacterial physiology, such as the distribution of BFM torque in a population or its dependence on the chemical environment, as well as other properties of biological relevance.

## Methods

### Stock solutions

For all stock solutions, chemicals were dissolved in deionized (DI) water with a resistivity of 18 MΩ cm from a water purification system (Milli-Q EQ 7000). A stock solution of 5% w/v F 108 (Sigma-Aldrich Synperonic F 108 surfactant, molecular weight (MW) of 14,600) was prepared by dissolving 50 g of F 108 in 1 l of DI water. A stock solution of 0.1 M potassium phosphate buffer (pH 8), which was used to prepare the motility medium, was prepared by dissolving 16.28 g of K_2_HPO_4_ (Sigma-Aldrich, MW 174.2) and 0.887 g of KH_2_PO_4_ (Sigma-Aldrich, MW 136.1) in 1 l of DI water. Finally, 0.5 M of ethylenediaminetetraacetic acid (EDTA) stock solution was prepared by dissolving 186.10 g of EDTA dihydrate (Sigma-Aldrich, MW 372.2) into 1 l of DI water. To dissolve the EDTA, the pH was adjusted to 8 using NaOH pellets. A stock solution of sterile ampicillin 100 mg ml^−1^ was purchased from Sigma-Aldrich. A 1 M L-serine solution was made by dissolving 1.05 g of L-serine powder (Sigma-Aldrich, MW 105.09 g mol^−1^) into 10 ml of DI water.

### *E. coli* growth

*E. coli* (strain MG1655) were labelled with GFP by in-house transformation using a standard electroporation protocol and a DNA plasmid containing both the GFP gene and an ampicillin-resistance gene.

Cultures were grown from single colonies on agar plates containing 100 μg ml^−^^1^ ampicillin. Cultures were grown overnight until saturation at 33 °C while being shaken at 200 rpm and in tryptone broth containing 10 g l^−1^ tryptone, and 5 g l^−1^ NaCl. The saturated culture was diluted 1:100 with fresh tryptone broth and grown at 33 °C until the optical density was 0.5, corresponding to the mid-exponential growth phase. Then, 1 ml of cells was centrifuged at 330*g* for 10 min until a pellet formed.

Cells were washed and gently resuspended in motility medium containing 1 mM potassium phosphate buffer (pH 8) and 0.1 mM EDTA (pH 8). This process was performed three times to ensure that the growth media had been sufficiently removed. In the final step, the optical density was remeasured and the final volume of motility medium was adjusted, yielding a concentrated sample of motile *E. coli* at an optical density of 1.

### Preparation of custom 3D-printed pucks

The 3D-printed pucks were printed using a commercial high-resolution two-photon-polymerization 3D-printing system (NanoOne, UpNano). The pucks were designed using OnShape CAD software. Glass coverslips (No. 1.5H high precision, 170 ± 5 μm, Zeiss) were cleaned via 10-min sonication in 1% Hellmanex solution, followed by 10-min sonication in DI water and finally by plasma-cleaning for 10 min. A 50 × 50 array of pucks was printed directly onto a glass coverslip (‘bottom-up’ printing mode) using a ×40 objective (numerical aperture 1.4 and working distance 130 μm) and UpBrix printing resin and applying standard fine-resolution printing parameters. Leftover resin was cleaned off with a 10-min soak in PGMEA solution ((1-methyl-2-propyl) acetate, Sigma-Aldrich), followed by a 2-min soak in 2-propanol (99.5% pure, Sigma-Aldrich).

Finally, the pucks were transferred to a 50-ml Falcon tube containing 5% F 108 stock solution and sonicated for 10 min to detach them from the glass coverslip. The coverslip was subsequently removed, and the pucks were allowed to sediment to the bottom. Finally, 1 ml was pipetted from the bottom of the tube, yielding a concentrated suspension of pucks dispersed in 5% F 108 surfactant. This process is illustrated in Supplementary Fig. 1.

### Pucks in solution

The concentrated solution of *E. coli* suspended in motility media was first diluted to the desired concentration, up to a maximum of *ρ* = 6 × 10^8^ cells per millilitre. L-serine stock solution (1 M) was added to enhance *E. coli* motility to a final concentration of 50 mM. Finally, pucks suspended in 5% F 108 surfactant were added to a final concentration of 0.25% F 108. The solution was confined in a 3 mm × 0.3 mm × 50 mm rectangular glass capillary and placed on a glass slide sealed with a wax pen. The glass capillary had previously been cleaned by sonication in 1% Hellmanex solution and washed by sonication in DI water.

### Reporting summary

Further information on research design is available in the Nature Portfolio Reporting Summary linked to this article.

## Online content

Any methods, additional references, Nature Portfolio reporting summaries, source data, extended data, supplementary information, acknowledgements, peer review information; details of author contributions and competing interests; and statements of data and code availability are available at 10.1038/s41567-026-03189-4.

## Supplementary information

Supplementary Information Captions for videos, and Supplementary Text, Figs. 1–8 and Table 1.

Reporting Summary

Supplementary Video 1 Dynamics of a puck without a channel. The video displays a 5-min (300-s) time-lapse recording of a puck without a channel,immersed in a bath of motile *E. coli* of concentration *ρ*_B_ = 6 × 10^8^ cells per millilitre. The puck rotates slowly in the clockwisedirection as *E. coli* collide with the perimeter. The video has been sped up ×10 real time. Scale bar: 20 μm.

Supplementary Video 2 Dynamics of a puck with four closed chambers. The video displays a puck with four chambers, each containing a singlebacterium. The puck rotates persistently in the clockwise direction. The real time of the video is 86 s. The video has been spedup ×4. Scale bar: 20 μm.

Supplementary Video 3 A single *E. coli* swimming through a channel. The video displays a single *E. coli* swimming through a 2 μm × 2 μm channel insidea puck. As the *E. coli* enters the channel, the puck initially rotates clockwise and then reverses direction as the *E. coli* exits. Thevideo plays in real time (4 s). Scale bar: 20 μm.

Supplementary Video 4 Collection of six pucks, each with four closed channels. The video displays the dynamics of six pucks, each with four closedchannels, in a bacterial bath. The pucks rotate rapidly, up to 10 rpm. The video exemplifies a way to investigate chiral fluids ofspinners. Pucks have radii of 10 μm. The real time of the video is 250 s, and it has been sped up.

## Data Availability

The datasets generated and analysed during the current study are openly available via Zenodo at 10.5281/zenodo.15236674 (ref. ^32^). All data are released under the CC-BY 4.0 licence. For any further questions about data access or reuse, please contact the corresponding author.
