# Supplementary material for: The hydrodynamic torque dipole from rotary bacterial flagella powers symmetric discs
Source: Nat Phys. 2026 Mar 27;22(4):620–7. doi: 10.1038/s41567-026-03189-4 (PMC13086583; doi:10.1038/s41567-026-03189-4)
Supplement: Supplementary file 1 — Captions for videos, and Supplementary Text, Figs. 1–8 and Table 1. [file 41567_2026_3189_MOESM1_ESM.pdf]

nature physics

Supplementary information

https://doi.org/10.1038/s41567-026-03189-4

The hydrodynamic torque dipole from rotary bacterial flagella powers symmetric discs

In the format provided by the authors and unedited

Contents

1 Description of movie materials **S2**

2 Supplementary Text **S3**

2.1 Supplementary Section: Experiments S3

2.1.1 Observation of puck dynamics S3

2.1.2 Differential Dynamic Microscopy S3

2.1.3 Observation of single swimmer passing through channel S3

2.1.4 Image Processing - Trajectory of pucks S4

2.1.5 Image Processing - *E. coli* swimming through the channel S4

2.1.6 Rotational diffusion of puck in thermal bath S5

2.1.7 Observation of puck with chamber S5

2.2 Supplementary Section: Collisions from clockwise swimming *E. coli* rotate symmetric discs S6

2.3 Supplementary Section: Simulations S7

3 Supplementary Figures and Table **S8**

Fig. S1: 3D printing, dispersion of pucks, and experimental setup S9

Fig. S2: Trajectories of *E. coli* swimming near a no-slip boundary S10

Fig. S3: Mean Squared Angular Displacement of pucks without a channel S11

Fig. S4: Rotational dynamics of a puck in thermal bath S12

Fig. S5: Single swimming bacteria crossing a puck with a rectangular channel S13

Fig. S6: Swimming bacteria crossing a puck with channel in opposite directions S14

Fig. S7: Comparison of correlation of the maximal rotation $\Delta\Theta_{max}$ of the pucks with statistical or dynamical properties of *E. coli* S15

Fig. S8: Enhancement χ of the torque in a closed channel as a function of the distance d from the wall S16

Table S1: Choice of parameters for simulations S17

1 Description of movie materials

Movie S1. Dynamics of a puck without a channel Movie S1 displays a 5 minute (300s) time-lapse of a puck without a channel, immersed in a bath of motile *E. coli* of concentration $\rho_B = 6 \times 10^8$ cells/mL. The puck rotates slowly in the clockwise direction as *E. coli* collide with the perimeter. Movie S1 is sped up 10x real time; scale bar 20 µm.

Movie S2. Dynamics of a puck with four closed chambers Movie S2 displays a puck with 4 chambers, each containing a single bacterium. The puck rotates persistently in the clockwise direction. Real time of the video is 86s. Movie S2 is sped up 4x; scale bar 20 µm.

Movie S3. A single *E. coli* swimming through a channel Movie S3 displays a single *E. coli* swimming through a 2 µm x 2 µm channel inside a puck. As the *E. coli* enters the channel, the puck initially rotates clockwise, and then reverses direction as the *E. coli* exits. Movie S3 plays at real time (4s); scale bar 20 µm.

Movie S4. Collection of 6 pucks, each with four closed channels Movie S4 displays the dynamics of 6 pucks, each with 4 closed channels, in a bacterial bath. The pucks rotate rapidly, up to 10RPM. Movie S4 exemplifies a route to investigate chiral fluids of spinners. Pucks are 10 µm radii. Real time of the video is 250s. Movie S4 is sped up 4x.

2 Supplementary Text

2.1 Supplementary Section: Experiments

2.1.1 Observation of puck dynamics

The glass capillary is observed on a Nikon TI-2 Eclipse microscope equipped with a Crest Optics X-Light spinning disk system, Lumencore Celesta Light Engine laser source, and a 100x Nikon Objective (Oil immersion, NA=1.45), focused on the bottom plane of the sample. 3000 frames at 10 fps are captured for each puck using spinning disk confocal microscopy; the sample is excited with a 488nm laser and emitted light passes through a multiband filter set from AVR Optics (BrightLine filter set, Part Number: Celesta- DA/FI/TR/Cy5/Cy7-A); both the pucks and *E. coli* are fluorescent at this wavelength. Images are captured using Prime 95B sCmos cameras from Teledyne Photometrics; a single camera is used for these experiments where the sample is excited using one light source. The observation is repeated for multiple pucks in the same capillary; We repeat the experiments with 4, 7, and 6 replicates for the radius 20, 10 and 5 µm pucks, respectively.

2.1.2 Differential Dynamic Microscopy

Differential Dynamic Microscopy (DDM) (*1,2*) is used to quantify the speed of the *E. coli* in videos used in Figure 1. Fluorescent images of both pucks and GFP-labeled *E. coli* are split into 500x500 pixel quadrants and analyzed independently, providing 4 measurements for each video; to extract the dynamics of only the *E. coli*, parameters are analyzed within a narrow region of reciprocal space q ($0.5 < q < 1.5$). The analysis is repeated for videos of different pucks, and averages are taken over pucks with the same radius. For each puck (R = 5, 10, 20 µm), the average speed of *E. coli* falls within the range of 14 ± 1 µm/s.

2.1.3 Observation of single swimmer passing through channel

To capture the dynamics of a single swimming going through the puck with a channel, we capture multiple 3000 frames videos at 10 fps; the videos are manually segmented into 40 frame clips which capture a single swimmer entering the channel. Videos are taken using excitation at both 488nm and 640nm; both the puck and the *E. coli* are fluorescent at 488nm, and only the puck is

fluorescent at 640nm. A beam splitter at 525nm separates the emitted light from the sample to two separate Prime 95B sCmos cameras (Teledyne Photometrics). These images are processed later to independently track the puck and location of *E. coli* in the channel.

2.1.4 Image Processing - Trajectory of pucks

To capture the angle of the pucks, we developed a tracking software in Python. In each frame, we extract the center of mass of the puck and the location of the small dot by applying a Difference of Gaussian filter; the radius of the filter is adjusted to detect either the puck or the small dot. A line is drawn between these two points, and the angle of this line with respect to the horizontal gives the orientation of the puck, as seen in the inset in Fig. 1A. The angular speed of the pucks is given by a linear regression between this orientation and time. To quantify the rotational diffusion of the pucks, we rotate the trajectory into this rotating frame of reference and calculate the Mean Squared Angular Displacement (MSAD); the rotational diffusion is given by a linear regression between MSAD and time, up to 1.5s [Fig. S3].

2.1.5 Image Processing - *E. coli* swimming through the channel

For the experiments where a single *E. coli* swims through the channel, we first track the puck using the Difference of Gaussian filter described previously. We track the orientation of the puck using only light greater than 525nm (red channel), to decouple locating of the center of mass of the puck from any light emitted from the *E. coli* as it swims through the channel. Next, we rotate the image into the frame of reference of the puck, such that the channel is placed horizontally. The *E. coli* is tracked using emitted light less than 525nm (green channel). We convolve the image with a Difference of Gaussian filter of size equal to the width of the *E. coli* and apply a binary mask to the image, revealing only the contents of the channel. Finally, the image is binarized; the length of the major axis of the binary region is taken as the size of the cell (ℓ_B); the orientation of the major axis with respect to the horizontal is taken as the angle of the *E. coli* inside the channel (Φ_e). For images where the *E. coli* cannot be effectively identified using the image processing techniques described above, the position cell body is located by clicking on the image. Finally, we track the center of the cell body from frame to frame, and extract a speed for each *E. coli*, as displayed in Figure 2A.

In Figure S7, we display data for 12 individual instances where a single *E. coli* runs through the

puck. For each instance, we track 4 statistics to describe the event: $\Delta\Theta_{max}$, the maximum change in angle of the puck over the timeframe where the *E. coli* is inside the channel; U_s, the velocity of the *E. coli*; $\langle\Phi_e\rangle$, the average angle of the *E. coli* cell body with respect to the channel; and ℓ_B, the length of the *E. coli* cell body. In Figure S7A we display a correlation matrix between these statistics; we find that $\Delta\Theta_{max}$ and ℓ_B are strongly correlated. In Figure S7B and Figure S7C, we plot $\Delta\Theta_{max}$ vs U_s and $\langle\Phi_e\rangle$, respectively. These variables do not display a strong correlation. Finally, in Figure S7D we plot U_s and ℓ_B; these variables do not display a strong correlation.

2.1.6 Rotational diffusion of puck in thermal bath

We measure the rotational mobility of the 10 µm puck by tracking the orientation of the puck in a thermal bath ($\rho_B = 0$). The experimental conditions are otherwise exactly identical to experiments including the bacterial bath; the pucks are suspended in Motility Media, 0.25% F-108 surfactant and 50mM L-serine, and sealed in a glass capillary. Because the rotation is very slow for an object of 10 µm radius, acquisition is performed over the course of hours. The puck is observed for 10,000 seconds at a frame rate 0.1 fps (1000 frames) using confocal microscopy. Figure S4A displays the angle of the puck as a function of time; this data is used to calculate an average Mean Squared Angular Displacement ($\langle\Delta\Theta^2\rangle$) at timesteps up to Δt = 1000s, as displayed in Figure S4B. A rotational diffusion coefficient (D_Θ) for the puck is determined by a linear fit between $\langle\Delta\Theta^2\rangle$ and Δt. We find $D_\Theta = 6 \times 10^{-5} \pm 1 \times 10^{-5}$ rad^2/s. The mobility of the puck is determined using the Stokes-Einstein relation:

$$D_\Theta = M_\Theta k_B T,$$

with M_Θ the rotational mobility, k_B the Boltzmann constant and T the temperature.

2.1.7 Observation of puck with chamber

In Figure 4, we investigate the dynamics of pucks with 4 closed chambers. These pucks are 3D printed, as described previously, and dispersed in solution containing Motility Medium, 0.25% F-08, and 50mM L-serine, and motile GFP-labeled *E. coli* (identical to previous experiments). The concentration of *E. coli* is adjusted to 1×10^8 cells/mL. We capture movies at 20 fps using the same

100x Nikon Objective and Nikon spinning disk confocal microscope described in previous section "Observation of puck dynamics". Cell body lengths are measured directly from images, using the Nikon Elements software.

2.2 Supplementary Section: Collisions from clockwise swimming *E. coli* rotate symmetric discs

In this section, we compare our experimental observations [Fig. 1C] of clockwise rotation of a symmetric disc in a suspension of swimming bacteria with a toy model, previously introduced in (*3*) [cf Fig. S5 and SI-Sec 2.3 in (*3*)].

We first recapitulate the model and then compare its predictions with our experimental results. In brief, straight swimmers colliding onto a disc exert a vanishing torque by symmetry. In contrast, this toy model predicts that the curvature of the circular clockwise trajectories of swimming bacteria leads to asymmetric collisions and subsequent persistent rotation. The tangential force exerted by a bacterium on the perimeter of the disc is $F_{\|}^{0} \propto \phi_e F_s$, where $\phi_e \sim \ell_B/R_c$ captures the asymmetry of collisions that arises from the curved trajectories of the bacteria, where ℓ_B is the length of the body of the *E. coli*, R_c is the radius of curvature of the trajectories, and F_s is the swim force of the bacterium, as discussed in the main text. The total effective tangential force exerted by the bacterial bath onto a disc is obtained by accounting for the number of bacteria $\tilde{N}$ colliding on the puck at any given time: $F_{\|}^{\text{eff}} \sim \tilde{N} F_{\|}^{0}$, where $\tilde{N} \sim \gamma\tau$ with γ the frequency of collisions of bacteria with the discs and τ the residency time of the bacterium on the disc (or duration of collision). To summarize:

$$F_{\|}^{\text{eff}} \sim (\ell_B/R_c) F_s \gamma\tau.$$

We now estimate these parameters in our experiments, e.g. [Movie S1, Fig. 1A-C], where we observe the clockwise rotation of pucks only through the collisions of *E. coli* with the perimeter of the disc. Following experimental measurements of the radius of curvature of *E. coli* trajectories [Fig. S2 (*3*)] and body length, we estimate $\phi_e \sim 0.1$. Next, we provide estimates from manual analysis of the time-lapse imaging for the frequency of collision $\gamma \sim 10\,\text{s}^{-1}$ and duration of collision $\tau \sim 0.3$s (lasting for 2 to 4 frames in the experiment). The estimates for the frequency of collision are corroborated by image analysis of Movie S1, determining the average number of *E. coli* in each frame, located less than 13 µm from the center of the puck, as 2.4 ± 1.4. Using $F_s \sim 0.2$ pN, the

pushing force of an *E. coli* as measured experimentally in (*4*), along with the numerical estimates above, leads to $F_{\parallel}^{\text{eff}} \sim 0.06\,\text{pN}$. Following, we estimate the expected rotation rate (ω) of a puck due to collisions with *E. coli* as:

$$\omega = M_{\Theta} F_{\parallel}^{\text{eff}} R,$$

with M_{Θ} the rotational mobility, as measured in experiments [Table.S1], and R the lever arm and radius of the puck ($R = 10\,\mu\text{m}$). This yields a value of $\omega \sim 10^{-2}$ rad/s, consistent with reported measurements in Figure 1C.

2.3 Supplementary Section: Simulations

We solve for the flow and traction fields induced by a point torque $\mathbf{\Gamma}$ inside an infinite channel using the boundary element method. By linearity of the Stokes equations Eq. (1), the velocity $\mathbf{U}$ in the fluid can be decomposed as a sum of two contributions: the flow $\mathbf{U}^t$ due to a point torque in free space in the absence of confinement, and a correction $\mathbf{U}^c$ calculated to satisfy the no-slip condition on the walls,

$$\mathbf{U}(\mathbf{r}) = \mathbf{U}^t(\mathbf{r}) + \mathbf{U}^c(\mathbf{r}). \tag{S1}$$

The first contribution is simply given by a rotlet flow,

$$\mathbf{U}^t(\mathbf{r}) = \frac{1}{8\pi\mu}\frac{\mathbf{\Gamma}\times\mathbf{r}}{|\mathbf{r}|^3}, \tag{S2}$$

where the position vector $\mathbf{r}$ points from the location of the singularity. The correction $\mathbf{U}^c(\mathbf{r})$ satisfies the homogeneous Stokes equations $\mu\nabla^2\mathbf{U}^c - \nabla P^c = \mathbf{0}$ and $\nabla\cdot\mathbf{U}^c = 0$, subject to the Neumann boundary condition $\mathbf{U}^c(\mathbf{r}) = -\mathbf{U}^t(\mathbf{r})$ for points $\mathbf{r}$ on the channel walls S, which ensures that the total flow field $\mathbf{U}(\mathbf{r})$ satisfies the no-slip condition. We solve for the correction $\mathbf{U}^c$ numerically using a single-layer boundary integral representation (*5*),

$$\mathbf{U}^c(\mathbf{r}) = \frac{1}{8\pi\mu}\int_S \mathbf{G}(\mathbf{r}-\mathbf{r}_0)\cdot\mathbf{q}(\mathbf{r}_0)\,\mathrm{d}S(\mathbf{r}_0), \tag{S3}$$

where $\mathbf{G}(\mathbf{r}) = (\mathbf{I}+\hat{\mathbf{r}}\hat{\mathbf{r}})/|\mathbf{r}|$ is the Oseen tensor, and $\mathbf{q}(\mathbf{r})$ is the unknown single-layer density defined on the domain boundaries. Evaluating Eq. (S3) on the channel walls and applying the boundary condition on $\mathbf{U}^c$ yields an integral equation for the unknown single-layer density $\mathbf{q}$, which we solve numerically after discretizing the channel walls with rectangular elements. The domain of

integration is truncated in the $\pm x$ direction at a distance chosen large enough to ensure convergence of the flow field, and $N = 1600$ elements are used in the calculations. After obtaining $\mathbf{q}(\mathbf{r})$ on the walls, we reconstruct the flow field inside the channel using Eq. (S3), and differentiate it numerically to obtain the surface traction $\mathbf{t}(\mathbf{r})$ entering Eq. (3) for the torque on the puck.

In the case of a closed chamber [Fig. 4], the flow solution above can be modified to satisfy the no-slip condition at the channel end as well. Assuming the channel is closed by a flat wall, the flow solution obtained above is easily modified using the method of images, by placing a counter-rotating image torque $-\mathbf{\Gamma}$ on the other side of the wall (*6*). The effect of the wall is to reduce the magnitude of the traction induced by the rotlet, with a modified value of Λ in Eq. (5) that now depends on the distance d from the rotlet to the channel end, and that we denote by Λ^d.

When the *E. coli* is inside the chamber, the net torque on the puck resulting from the two rotlets is modified as

$$\Gamma = -\Lambda^d \left(\frac{x_1 - x_C}{W}\right)\Gamma_M + \Lambda^{d+\ell_D}\left(\frac{x_2 - x_C}{W}\right)\Gamma_M, \tag{S4}$$

where d is the distance from the wall to the first rotlet, and $d + \ell_D$ is the distance to the second rotlet. Also, $x_1 - x_C = d + d_C$, where d_C is the offset from the channel end to the center of the puck, which is provided by the experimental design and is on the order of 1 µm. We find that Λ^d is a rapidly decaying function of d, and given the value of ℓ_D, $\Lambda^{d+\ell_D} \approx \Lambda$ in all cases. Because the effect of the wall is to decrease the magnitude of the traction field induced by the first rotlet, it results in an enhancement of the net torque on the puck. The factor that captures the enhancement of the torque with respect to the value in open channels is given by the ratio of Eq. (S4) and Eq. (6) and simplifies to

$$\chi = \frac{\Gamma}{-\Lambda\left(\frac{\ell_D}{W}\right)\Gamma_M} = \left[1 + \frac{(d + d_C)}{\ell_D}\left(1 - \frac{\Lambda^d}{\Lambda}\right)\right]. \tag{S5}$$

The dependence of this ratio on the distance d is plotted in Fig. S8, and shows an enhancement of $\sim 0 - 15\%$. The precise value of d is unknown, but we can estimate it to be on the order of 0.5 µm based on the channel geometry if we approximate the *E. coli* body as a cylinder of radius 0.5 µm. For this value of d, our model predicts a torque enhancement of $\sim 10\%$.

3 Supplementary Figures and Table

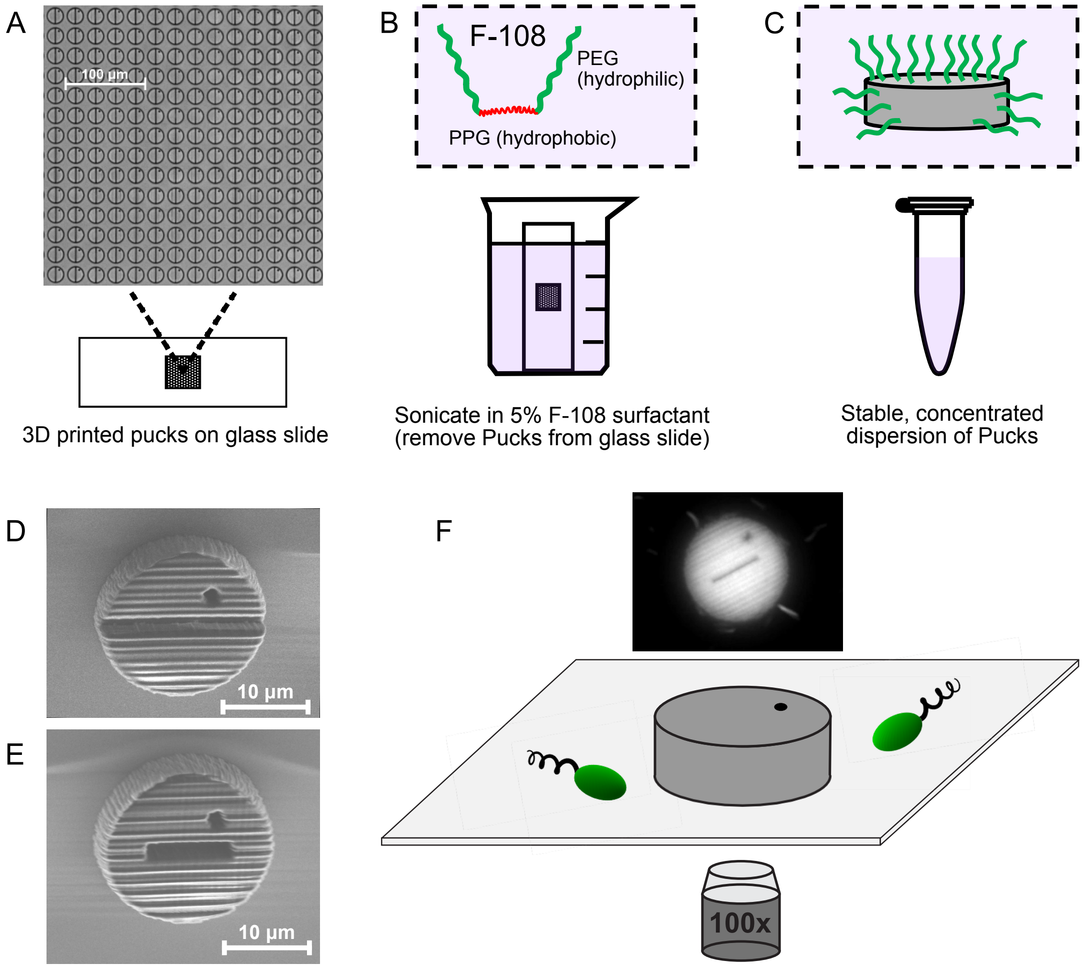

Figure S1: **3D printing, dispersion of pucks, and experimental setup** **(A)** Pucks are initially 3D printed on a glass coverslip, using 2-Photon-Polymerization (2PP) technique, in 50x50 arrays. **(B)** To remove the pucks from the glass, the coverslip is placed in a 50mL Falcon tube containing 5% F-108 surfactant, and sonicated. F-108 is a tri-block copolymer, containing two hydrophilic sections (PEG) and a hydrophobic section in the middle (PPG). **(C)** The glass coverslip is subsequently removed, and pucks sediment. Finally, a concentrated dispersion of pucks is obtained by pipetting the bottom 1mL of solution into an eppendorf tube. **(D-E)**. Scanning Electron Microscopy (SEM) images of the pucks used in this work. The stripes visible on the images reflect the voxel size during 2PP and are ~ 100nm in height, measured independently with an AFM. **(D)** SEM image of a puck with a channel. Scale bar 10 µm. **(E)** SEM image of a puck without a channel. Scale bar 10 µm. **(F)** Schematic representation of the experiment, where pucks are immersed in a bath of swimming *E. coli* bacteria and imaged with fluorescence microscopy using a 100x objective. (Top picture) Fluorescence microscopy image of a puck.

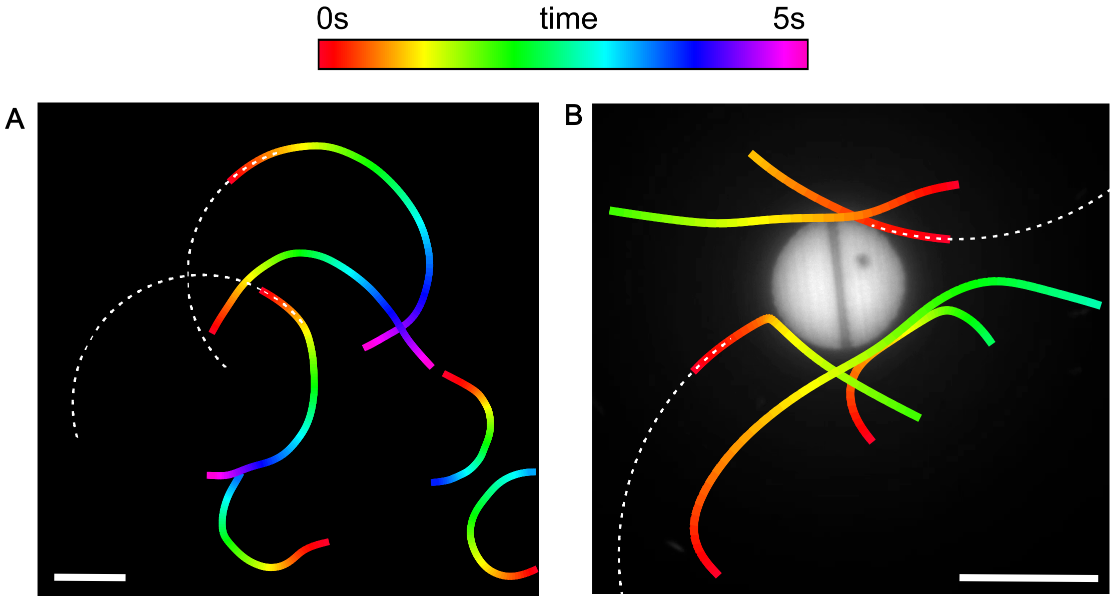

Figure S2: **Trajectories of *E. coli* swimming near a no-slip boundary (A)** *E. coli* swim in clockwise circular trajectories when swimming above a glass interface. Each colored line represents the trajectory of a single *E. coli*; time is indicated by the colorbar. The white dashed curves represent a circle of radius 40 µm. Scale bar 20 µm. **(B)** Each colored line represents the trajectory of a single *E. coli* which collides with the exterior of the puck. The *E. coli* swim in clockwise circular trajectories and scatter off the exterior of the puck. Scale bar 20 µm.

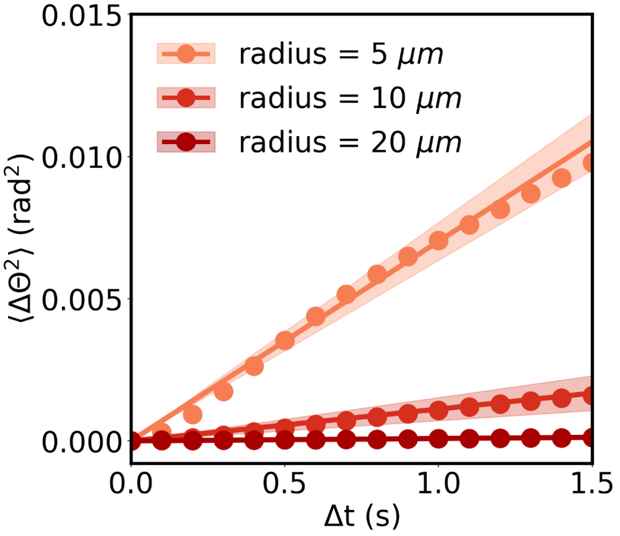

Figure S3: **Mean Squared Angular Displacement of pucks without a channel** Mean Squared Angular Displacement ($\langle \Delta\Theta^2 \rangle$) is calculated by first moving the angular trajectory of the puck into a rotating reference frame, equal to its average angular velocity; $\langle \Delta\Theta^2 \rangle$ is calculated by taking the average change in angle of the puck at time steps (Δt), ranging from 0.1 to 1.5 s. The rotational diffusivity of the puck is quantified via a linear fit between $\langle \Delta\Theta^2 \rangle$ and Δt. In the above figure, dots represent the trajectory of a single puck, while solid lines and shaded regions represent the average and standard deviation over all pucks of that size. We capture 6, 7, and 4 pucks of sizes $R = 5$, 10 and 20 μm, respectively.

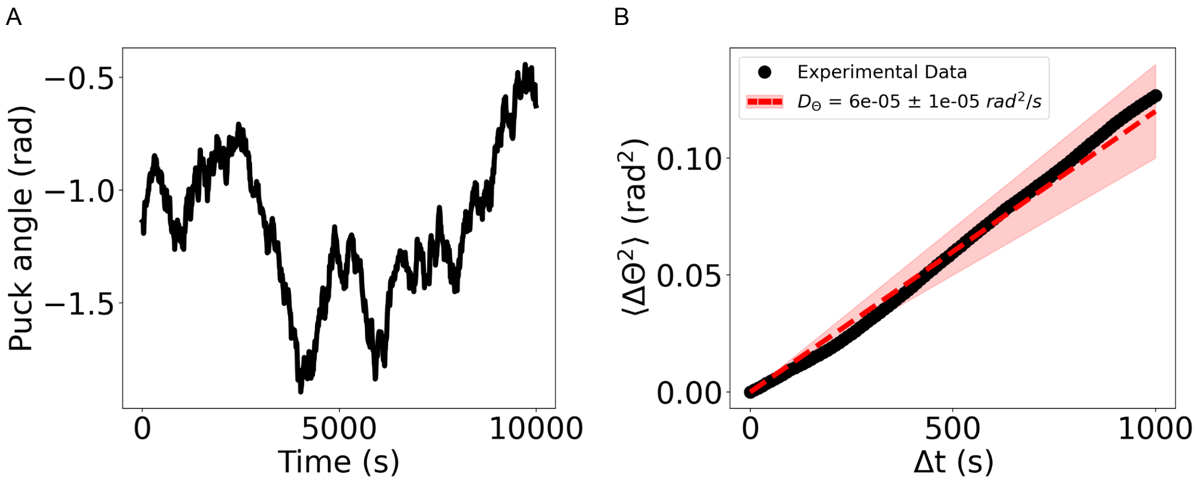

Figure S4: **Rotational dynamics of a puck in thermal bath (A)** The orientation of a single puck (R = 10 µm) in a thermal bath is tracked for 10,000 seconds, using a frame rate of 0.1fps. **(B)** Average Mean Squared Angular Displacement ($\langle \Delta\Theta^2 \rangle$) as a function of timestep (Δt) is calculated using the trajectory shown in **(A)**. A rotational diffusion coefficient for the puck (D_Θ) is extracted via linear fit $\langle \Delta\Theta^2 \rangle = 2D_\Theta \Delta t$, and visualized with the red dashed line; a confidence interval representing estimated standard measurement error is displayed in red shaded region. We determine the rotational diffusion coefficient to be $D_\Theta = (6 \pm 1) \times 10^{-5}$ rad^2/s.

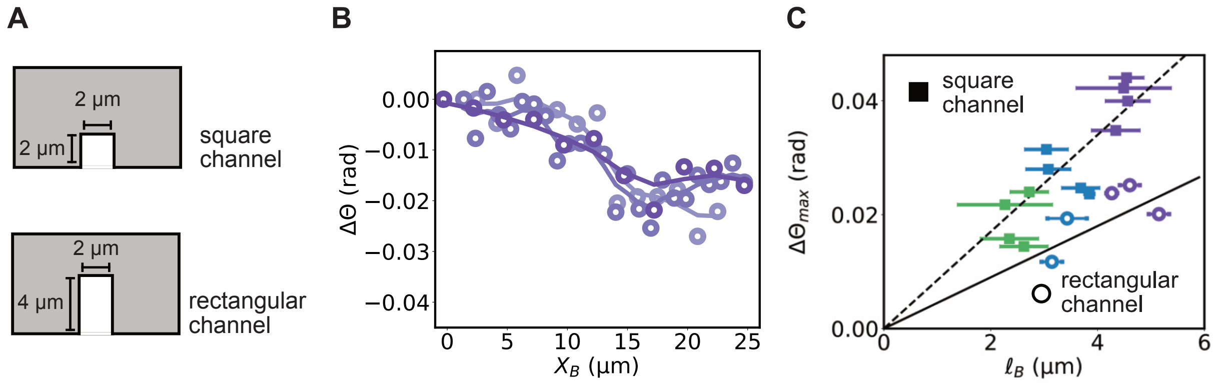

Figure S5: **Single swimming bacteria crossing a puck with a rectangular channel (A)** We perform additional experiments, investigating the effect of varying the geometry of the channel. The experiments presented in this figure utilize a rectangular channel of width 2 µm and height 4 µm, as opposed to the square channel (width 2 µm and height 2 µm), presented in the main text. **(B)** Rotation of the puck with a rectangular channel, as *E. coli* swim through the channel. The dynamics of the puck present the characteristic down-up curve, discussed previously, however the depth of the minimum $\Delta\Theta_{max}$ is reduced compared to trajectories displayed in Figure 2C. **(C)** Comparison of the depth $\Delta\Theta_{max}$ of the down-up shape for different geometries. The data from Figure 2E (solid squares), using a square (width 2 µm and height 2 µm) channel, are significantly higher than the experiments using a rectangular channel (width 2 µm and height 4 µm) with double the height (open circles). The measurements for $\Delta\Theta_{max}$ increase linearly with ℓ_B, as predicted by the hydrodynamic model. The reduction of the slope nears 50% for the taller channels, highlighting the salient effect of confinement to transmit the torque dipole of the bacteria to the puck.

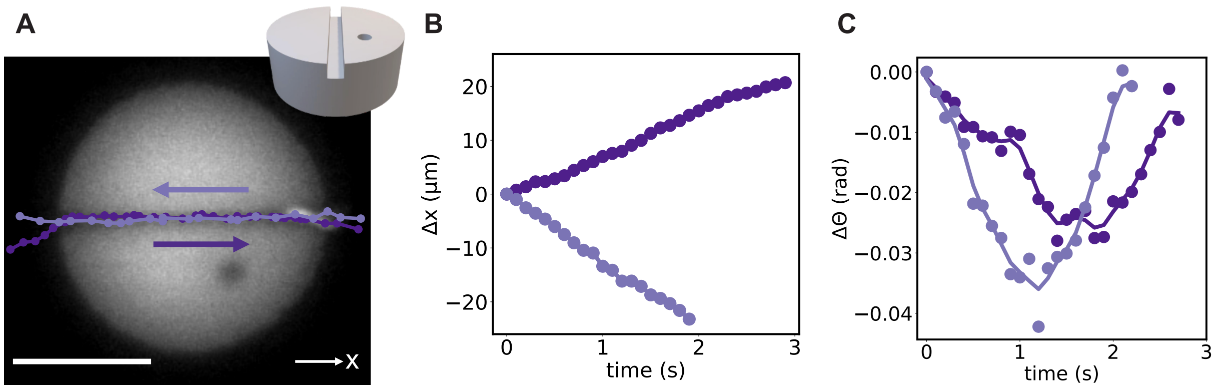

Figure S6: **Swimming bacteria crossing a puck with channel in opposite directions** **(A)** We display 2 trajectories of *E. coli* swimming through the channel of a puck. The *E. coli* enter from opposite sides of the puck, with the direction of travel indicated by an arrow. A 3D representation of the puck is included in the top right. Scale bar 10 µm. **(B)** Evolution of the position of the *E. coli* as they cross the puck through the channel. The opposite slopes indicate that the *E. coli* enter from opposite sides of the channel, and move with constant speed without stopping or reorienting inside the channel. **(C)** The dynamics of the angle of the puck, $\Delta\Theta(t)$, remains qualitatively the same, irrespective of the entry side and direction of crossing of the swimming bacterium in the channel; the puck first rotates clockwise as the *E. coli* enters, and rotates counter-clockwise as the *E. coli* exits, giving the characteristic down-up curve.

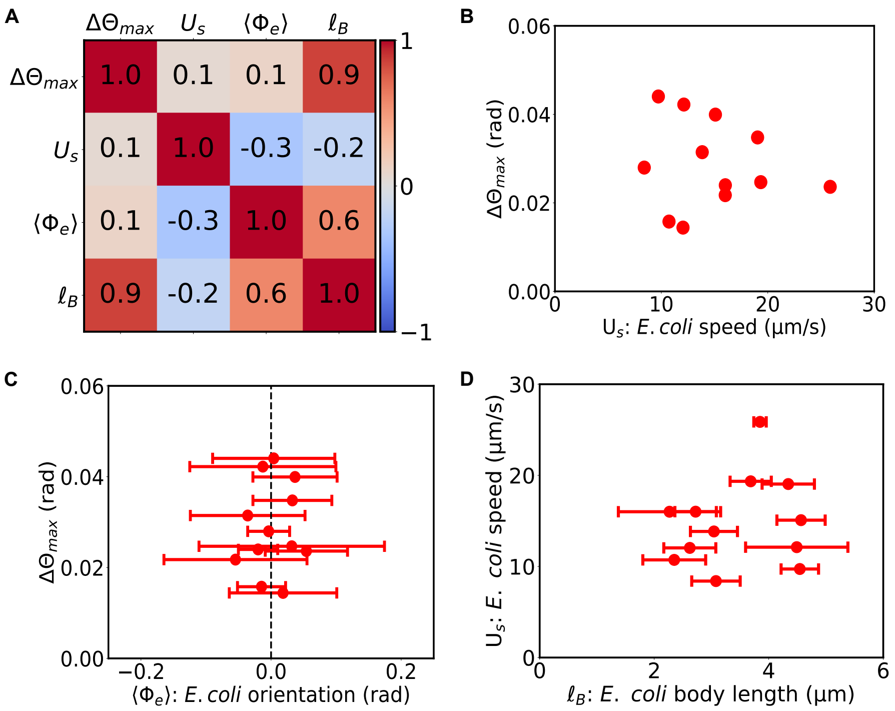

Figure S7: **Comparison of correlation of the maximal rotation $\Delta\Theta_{max}$ of the pucks [see main text] with statistical or dynamical properties of *E. coli*.** In order to understand the mechanism of rotation of the puck, we identify parameters that intuitively could induce the rotation of the puck and investigate their correlation. Following past work that showed the rotation of microgears in bacterial baths due to collisions, we notably investigate the effect of the speed of the *E. coli* and their average orientation in the channel. As visible from (A), the correlation is weak, highlighting that our mechanism for rotating the pucks is different than previously reported. Remarkably, it correlates well with the length of the bacterium body, as explained by our model of torque dipoles [see main text]. **(A)** Each time an *E. coli* swims through the channel, the rotation of the puck is quantified using the maximum change in angle of the puck ($\Delta\Theta_{max}$) during the interval where *E. coli* is inside the channel. The motion of the *E. coli* is quantified using 3 statistics: its speed (U_s), its average angle with respect to the channel ($\langle\Phi_E\rangle$), and the length of the cell body (ℓ_B). The correlation matrix displays the Pearson correlation coefficient between such variables. **(B)** Maximum change in angle of the puck ($\Delta\Theta_{max}$) and *E. coli* speed (U_s); each dot represents a single *E. coli* swimming through the channel. No significant correlation is found between $\Delta\Theta_{max}$ and U_s. **(C)** Maximum change in angle of the puck ($\Delta\Theta_{max}$) and average angle of the *E. coli* with respect to the channel ($\langle\Phi_E\rangle$). No significant correlation is found between $\Delta\Theta_{max}$ and $\langle\Phi_E\rangle$. **(D)** *E. coli* speed (U_s) and length of the cell body (ℓ_B). No significant correlation is found between U_s and ℓ_B.

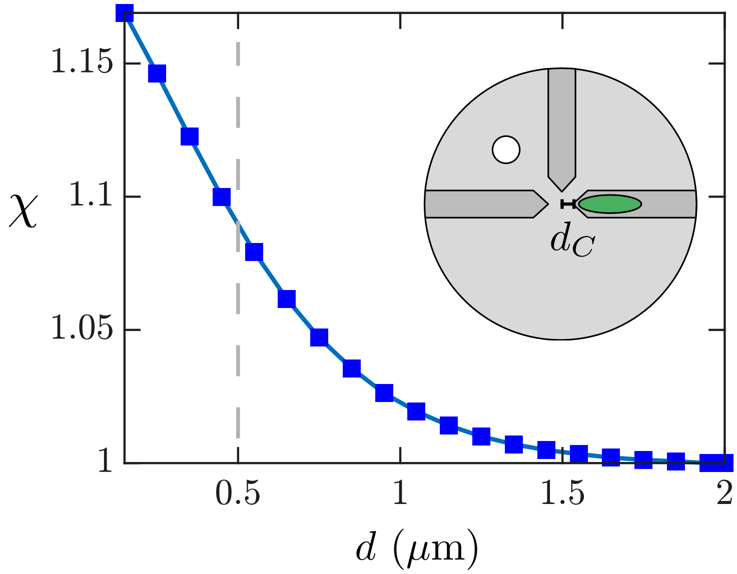

Figure S8: **Enhancement χ of the torque in a closed channel as a function of the distance d from the wall.** In a closed channel, the no-slip condition at the channel end results in a decrease in the traction induced by the first rotlet, and thus causes an increase in the net torque on the puck by a factor χ estimated in Eq. (S5). The distance d from the first rotlet to the wall is estimated to be $\sim 0.5\,\mu\text{m}$ in the experiment (vertical dashed line), corresponding to a torque enhancement of $\sim 10\%$.

Table S1: **Choice of parameters for simulations** The following table includes values used to fit our hydrodynamic simulations to the experimental work, notably in Fig. 3C and Fig. 4B.

**Choice of parameters**			
Variable	References	Range	Current work
Motor torque Γ_M	(*7*) (*8*) (*9*)	400-4500 pN-nm	1600 pN-nm
Stokesian mobility of the puck M_Θ	Supplemental material, [Fig. S4]	$[1.2,1.7]\times10^{-5}$ (sec-pN-nm)$^{-1}$	2×10^{-5} (sec-pN-nm)$^{-1}$
Swim speed of the bacterium U^s	[Fig. S7D], (*3*)	5-25 µm/s	15 µm/s
Puck radius R	Main Text [Fig. 1D]	5-20 µm	10 µm
Channel height H<br>Channel width W	Main Text<br>[Fig. 1D]	2 µm<br>2 µm	2 µm<br>2 µm
Rotlet dipole size ℓ_D	Main text, [Fig. S7D]	$\alpha\times$ cell body, $\alpha = 1.5$	1.5 ℓ_B

References and Notes

1. R. Cerbino, V. Trappe, Differential Dynamic Microscopy: Probing Wave Vector Dependent Dynamics with a Microscope. *Phys. Rev. Lett.* **100**, 188102 (2008), doi:10.1103/PhysRevLett.100.188102.

2. L. G. Wilson, *et al.*, Differential Dynamic Microscopy of Bacterial Motility. *Phys. Rev. Lett.* **106** (1), 018101 (2011), doi:10.1103/physrevlett.106.018101.

3. D. Grober, *et al.*, Unconventional colloidal aggregation in chiral bacterial baths. *Nature Physics* **19** (11), 1680–1688 (2023), doi:10.1038/s41567-023-02136-x.

4. G. Vizsnyiczai, *et al.*, Light controlled 3D micromotors powered by bacteria. *Nature Communications* **8** (1), 15974 (2017), doi:10.1038/ncomms15974

5. C. Pozrikidis, *Boundary Integral and Singularity Methods for Linearized Viscous Flow*, Cambridge Texts in Applied Mathematics (Cambridge University Press, Cambridge) (1992), doi:10.1017/CBO9780511624124.

6. J. R. Blake, A. T. Chwang, Fundamental singularities of viscous flow. Part I: The image system in the vicinity of a stationary no-slip boundary. *Journal of Engineering Mathematics* **8**, 23–29 (1974), doi:10.1007/BF02353701.

7. D. Das, E. Lauga, Computing the motor torque of Escherichia coli. *Soft matter* **14** (29), 5955–5967 (2018), doi:10.1039/C8SM00536B.

8. R. M. Berry, H. C. Berg, Absence of a barrier to backwards rotation of the bacterial flagellar motor demonstrated with optical tweezers. *Proceedings of the National Academy of Sciences* **94** (26), 14433–14437 (1997), doi:10.1073/pnas.94.26.14433.

9. K. A. Fahrner, W. S. Ryu, H. C. Berg, Bacterial flagellar switching under load. *Nature* **423** (6943), 938–938 (2003), doi:10.1038/423938a.
